# KinFams: De-Novo Classification of Protein Kinases Using CATH Functional Units

**DOI:** 10.3390/biom13020277

**Published:** 2023-02-02

**Authors:** Tolulope Adeyelu, Nicola Bordin, Vaishali P. Waman, Marta Sadlej, Ian Sillitoe, Aurelio A. Moya-Garcia, Christine A. Orengo

**Affiliations:** 1 Institute of Structural and Molecular Biology, University College London, London WC1E 6BT, UK; 2 Department of Comparative Biomedical Science, Louisiana State University, Baton Rouge, LA 70803, USA; 3 Departamento de Biología Molecular y Bioquímica, Universidad de Málaga, 29071 Málaga, Spain; 4 Laboratorio de Biología Molecular del Cáncer, Centro de Investigaciones Médico-Sanitarias (CIMES), Universidad de Málaga, 29071 Málaga, Spain

**Keywords:** protein kinases, functional families, KinFams, KinBase classification

## Abstract

Protein kinases are important targets for treating human disorders, and they are the second most targeted families after G-protein coupled receptors. Several resources provide classification of kinases into evolutionary families (based on sequence homology); however, very few systematically classify functional families (FunFams) comprising evolutionary relatives that share similar functional properties. We have developed the FunFam-MARC (Multidomain ARchitecture-based Clustering) protocol, which uses multi-domain architectures of protein kinases and specificity-determining residues for functional family classification. FunFam-MARC predicts 2210 kinase functional families (KinFams), which have increased functional coherence, in terms of EC annotations, compared to the widely used KinBase classification. Our protocol provides a comprehensive classification for kinase sequences from >10,000 organisms. We associate human KinFams with diseases and drugs and identify 28 druggable human KinFams, i.e., enriched in clinically approved drugs. Since relatives in the same druggable KinFam tend to be structurally conserved, including the drug-binding site, these KinFams may be valuable for shortlisting therapeutic targets. Information on the human KinFams and associated 3D structures from AlphaFold2 are provided via our CATH FTP website and Zenodo. This gives the domain structure representative of each KinFam together with information on any drug compounds available. For 32% of the KinFams, we provide information on highly conserved residue sites that may be associated with specificity.

## 1. Introduction

Protein kinases are enzymes involved in multiple cellular pathways. They catalyse the transfer of phosphate from a phosphate donor to the hydroxyl groups of acceptor molecules which can either be protein substrates, lipids or small molecules. Most kinases use ATP as their phosphate donor, however some use other donors, such as GTP, ADP, inorganic pyrophosphate (PPi) and others [1,2]. Through this phosphorylation process, the targets are covalently modified leading to the regulation of biological processes, such as the control of metabolism, transcription processes, cell division and movement, programmed cell death and several other signal transduction events in the cell. About 2% of the human genome encodes for protein kinases [2]. They are the second largest enzyme family and the fifth largest family of genes in humans, following zinc finger proteins, G-protein coupled receptors, immunoglobulins, and proteases [3]. Protein kinases can be broadly classified as either tyrosine kinases or serine/threonine kinases based on the specificity of the substrate they phosphorylate. 

**Figure 1:**
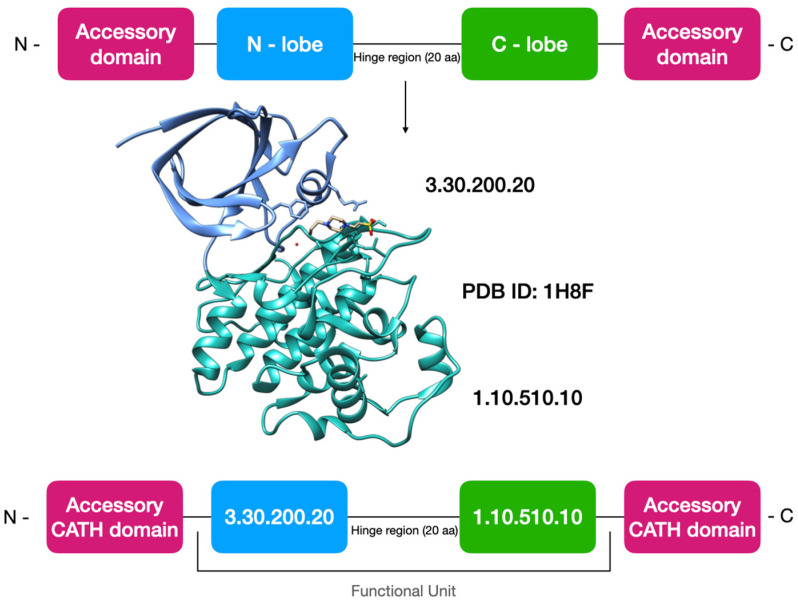
Schematic representing the generation of kinase functional units from the separate kinase domains in CATH. The 3D structure is shown using PDB ID:1H8F. The kinase N-lobe domain (blue box) is classified in the CATH 3.30.200.20 superfamily, while the C-lobe domain is classified in the CATH 1.10.510.10 superfamily.

The protein kinase catalytic domain is structurally conserved and comprises around 250 to 300 amino acid residues [4]. It contains two lobes (N- and -C) connected through a flexible hinge region with the active site in a cleft between the lobes, which together acts as a functional unit (see Figure 1). The smaller N-lobe contains the highly conserved C-helix [5]. The larger C-lobe is mainly α-helical and contains the helices called E and G in its conserved core. Other important structural motifs are the phosphate-binding loop and the activation loop (A-loop), which bind ATP and the peptide substrate, respectively [6]. Kinases display remarkable diversity in their primary sequences, substrate specificity, structure and the pathways associated with them. However, they share a great degree of similarity in their 3D structure and especially in their catalytic site where the ATP-binding cavity is found [7,8]. ATP binds in the cleft between the N and C lobes and therefore most kinase inhibitors interact with this region to perturb the binding of ATP.

Most kinase family classification systems derive from the seminal work by Hanks and Hunter [7] that uses the amino acid sequences of the catalytic domains, and which divides kinases into groups, families and subfamilies. In 1997, the Bourne group built on this work and included an additional dataset of ~1600 kinase sequences from the SwissProt and PIR resources [9]. They made their data available through the Protein Kinase Resource (PKR), which comprises nine groups, 81 families and 238 subfamilies [9]. This was one of the very first resources to make the kinase classification data available online together with structural annotations from the Protein Databank (PDB) and disease information from the OMIM database [9]. 

The currently most widely used standard classification system was later developed by Manning and colleagues in 2002 and made available via the KinBase resource [10]. Members within a KinBase group have a broad substrate site specificity; members within a family are grouped together based on sequence similarity and their biological function. Some of the families in KinBase are further subdivided into subfamilies based on finer sequence-level and functional similarity. To date, KinBase classifies protein kinases from 15 organisms, into 14 groups, 240 families and 339 subfamilies (according to the latest KinBase version 2014; kinase.com, accessed on 24 January 2023). The kinomes from the following 15 organisms are classified in KinBase–*H*. *sapiens*, *M*. *musculus*, *C. elegans*, *D. melanogaster*, *S. cerevisiae*, *D. discoideum* and *T. thermophila*, *A. queenslandica*, *M. brevicollis*, *C. cinerea*, *G. lamblia*, *L. major*, *T. vaginalis* and *S. moellendorffii*.

Several other studies subsequently used or expanded these kinase classification schemes (See Table 1). The Barton group used a multilevel hidden Markov model (HMM) library to map sequences from SwissProt (version 2004) for *H*. *sapiens*, *M*. *musculus*, *D*. *melanogaster*, *C*. *elegans*, *S*. *cerevisiae*, and *D*. *discoideum* and from 21 other additional eukaryotic species [11,12]. This data was made available through the Kinomer database, which provides only group-level classification based on KinBase version 2008 [13]. 

In 2004, the Srinivasan group developed the KinG database [14], the first database to include sequences from bacteria (total 27 species), archaea (total eight species) and plant species (*Arabidopsis thaliana*). These were mapped to families from Bourne’s Protein Kinase Resource (PKR, 9). Srinivasan and co-workers showed how information on other domains tethered to the kinase catalytic domain revealed outliers in classical kinase classifications that could be used to refine the classification [17]. They considered the composition of the kinase accessory domains and the organisation of these domains. Classification was refined manually using an alignment-free method to detect the similarity between sequences by assessing short amino acid sequence patterns and structural features outside the catalytic domain. Using this approach, they were able to detect outliers called “hybrid kinases” that had sequence regions associated with the catalytic domains matching a particular subfamily but regions outside the catalytic domain matching a different subfamily [17]. The standard classification approach using only the catalytic domain sequences would not have been adequate to capture these cases. KinG currently holds information on >2000 organisms (including eukaryotes, viruses and prokaryotes) and allows searches for kinases based on domain combinations [14].

Other integrated resources also exist [18,19,20]. In 2011, Ghosal et al. [15] developed the protein kinase ontology (ProKinO) framework for human kinases, which now also provides family annotations for 1321 species, by mapping sequences to KinBase (version 2012) and integrating data from COSMIC, UniProt and Reactome [15]. This framework has been used in the analysis of cancer-associated mutations [18] and recently to annotate dark kinases (i.e., experimentally uncharacterised) in humans [21,22]. A similar resource, KinHub, also provides annotations specific to human kinases (http://www.kinhub.org, accessed on 24 January 2023). KIDFamMap [19] provides a platform for accessing the kinase conformational types and functions to gain biological insights into the selectivity of human kinase inhibitors and mechanisms of action. 

These resources provide a rich source of annotations of existing families in KinBase or Protein Kinase Resource (PKR) (see Table 1), however most of them are based on KinBase and are not completely up to date with sequences from all organisms in UniProt (see Table 1). Since the Manning group developed KinBase, there has been a significant expansion in protein kinase sequences deposited in UniProt (https://www.uniprot.org/help/downloads [23], accessed on 24 January 2023) and other public sequence repositories. Whilst other large resources, such as Pfam ([24], https://pfam.xfam.org, accessed on 24 January 2023) and PANTHER ([25], http://www.pantherdb.org/, acceseed on 24 January 2023) classify these proteins into evolutionary families, they do not explicitly classify them into distinct functional families, i.e., comprising evolutionary relatives sharing similar functional properties. 

The CATH classification currently classifies the kinase functional unit into two separate domains corresponding to the N-lobe and the C-lobe, as they are distinct globular regions. Since both are required to provide the function, we have generated a new category of superfamily in CATH, corresponding to the kinase ‘functional unit’, which concatenates one or more domains contributing to the functional role of the protein. Subsequently our CATH-FunFam (functional family) resource uses automated approaches including agglomerative clustering and an entropy-based protocol, to segregate functionally distinct groups by implicit identification of specificity-determining positions (SDPs) and other functional sites [26]. CATH-FunFams have been endorsed in-silico [26,27] and by blind independent assessment in CAFA, in which CAFA-FunFams were recently highly ranked for prediction of molecular function [28]. 

In this study, we report the classification of kinase functional families (CATH-KinFams), from all kinase sequences available in UniProt (version 2018_02) using an improved FunFam classification method (FunFam-MARC). Our automated approach has allowed us to update information on sequences deposited in UniProt since the development of Protein Kinase Resource (PKR), KinBase, and other related kinase resources, and to identify new families (and subfamilies) and their relationship to families defined in Manning’s KinBase classification. 

The CATH FunFam-based protocol explicitly exploits information on the multi-domain architecture (MDAs) of protein kinases. Our automated classification protocol identifies a total of 2210 CATH-KinFams, the majority of which are observed to have high functional purity in terms of EC annotations. Since mutations in protein kinases have been recorded in several diseases especially cancer and kinases are a major therapeutic target, we also analyse our human-associated KinFams in the context of disease information and drugs, based on a CATH FunFam-based protocol developed earlier [29]. Our KinFam classification is currently the most comprehensive in terms of functional families and species and the data is available for download on Zenodo (https://zenodo.org/record/7575924, accessed on 24 January 2023), the CATH FTP site (ftp://orengoftp.biochem.ucl.ac.uk/kinfams, accessed on 24 January 2023) and will be made available via the CATH-FunVar website (https://funvar.cathdb.info/ [16], accessed on 24 January 2023),. The multi-domain-based functional family classification method designed for classification of the kinases, can be readily extended to other important classes of enzymes and drug targets.

## 2. Materials and Methods

### 2.1. Generating CATH-KinFams

CATH typically classifies the functional unit in protein kinases into two separate domains corresponding to the N- and C- lobes (or domains). These are represented as the CATH superfamilies 3.30.200.20 (N-domain) and 1.10.510.10 (C-domain), respectively (https://www.cathdb.info/, accessed on 24 January 2023). As the majority of the protein kinase inhibitors act at the hinge region between these two domains, we have created a new level within CATH to classify such ‘functional units’. This will clearly be valuable for enzymes and other proteins where the functional unit straddles more than one domain. In the context of the kinases, not only will it enable us to better understand the relationships between different kinases, but it will be essential for understanding kinase–drug interactions and enabling drug repurposing. The concept of a functional unit is illustrated in Figure 1 (illustrated using PDB ID: 1H8F).

### 2.2. Updating Kinase Domain Sequences in the CATH Family Classification and Generating the Kinase Functional Unit

The CATH kinase superfamilies were updated to include the most recent version of UniProt (UniProt release 2018_02). This was achieved by scanning UniProt sequences against the library of HMMs built from all CATH structural representatives using HMMer3 [30]. CATH-resolve-hits [31] was then used to identify significant matches to the kinase N- and C- domain superfamilies (CATH superfamilies ‘1.10.510.10’ and ‘3.30.200.20’, respectively) and to other CATH domains. Kinase functional units were constructed for each protein kinase by concatenating the domain sequences from the N- and C- lobe domains. We allowed a linker (up to 20 residues long) between the two lobes to ensure that we covered the complete kinase hinge region. The multi-domain architectures (MDA) (i.e., the order of the domains along the protein sequence, including the kinase functional unit and the additional domain partners) were determined using the CATH resolve-hits (CRH) protocol [31]. CRH uses an optimisation algorithm to resolve matches to the CATH HMM libraries and obtain a set of non-overlapping domain annotations for the sequence.

### 2.3. Running the FunFam-MARC Algorithm

**Figure 2:**
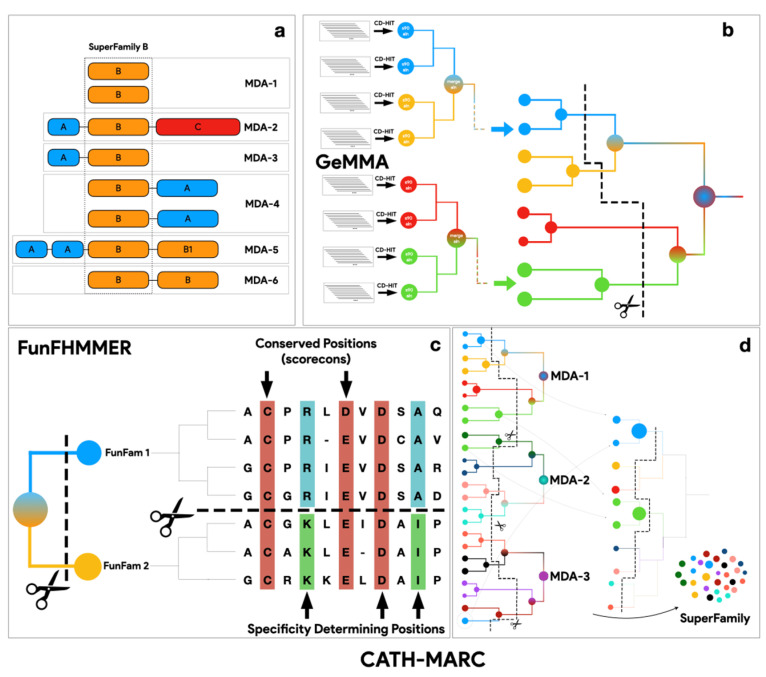
FunFam-MARC protocol. (**a**). FunFam-MARC approach based on multi domain architectures, (**b**). Overview of GeMMA/FunFHMMER protocol, (**c**). Example of FunFHMMER detection of specificity determining positions, (**d**). Multiple iterations of GeMMA/FunFHMMER with MDA pooling.

FunFam-MARC (multidomain architecture-based clustering) is a suite of protocols, as summarised in Figure 2. It first partitions the set of kinase functional unit sequences into subsets of sequences having the same MDA (i.e., the same domains in the same order in the protein sequence). Within each MDA partition, the sequences are clustered into 90% sequence identity clusters (S90) using CD-HIT [32]. These CD-HIT clusters are the starting point for the next step in FunFam-MARC which applies GeMMA [33] (see Figure 2a), a method for deriving a tree of sequence relationships in a protein superfamily. In the first step of GeMMA, S90 clusters are annotated with experimentally characterised GO terms (e.g., TAS) obtained using the UniProt API [34]. Since FunFam-MARC is computationally expensive, clusters having no experimental GO annotations are discarded. HHsuite is used to generate HMMs for each S90 cluster [35]. Subsequently, GeMMA applies agglomerative clustering by performing all against all HMM comparisons between clusters and then progressively merging clusters with the highest scores (see Figure 2a). This generates an input tree for FunFHMMER [27], a method that cuts the tree into clusters of functionally similar sequences. FunFHMMer traverses the tree from leaves to the root, cutting the tree where the branches comprise clusters with significant differences in function determining residues. These are identified by using GroupSim [36]; see Figure 2b) which detects differences in conservation patterns between equivalent residues in the combined multiple sequence alignment of the two clusters being considered. Functional determinants, i.e., Specificity-Determining Positions (SDPs) are identified as residues which are differentially conserved between FunFam clusters.

Once all MDAs have been processed, FunFam clusters from each MDA partition are pooled and form the starting clusters for a final run of GeMMA and FunFHMMer. Following this final iteration of tree building and segregation into clusters, the resulting clusters are the final kinase FunFams (subsequently referred to as KinFams). Finally, we scan the sequences from the experimentally uncharacterised S90 clusters against the final kinase FunFam HMMs to determine how close they are to functionally characterised FunFams to help guide the functional characterisation of these clusters. The FunFam-MARC protocol is illustrated in Figure 2.

### 2.4. Assessing the Functional Coherence of KinFams and KinBase Classifications Using the Enzyme Classification

We assessed the functional coherence of the Kinase FunFams (KinFams) and Kinase families by examining the agreement in experimental EC-annotations between sequence relatives in a given KinFam. That is, we determined whether relatives in each KinFam had the same or similar Enzyme Classification (EC) numbers. This is an established approach previously used to validate CATH FunFams [26,37]. The enzyme classification is a 4-digit numerical classification scheme based on the chemical reactions of enzymes [38]. The first digit describes the general type of reaction the enzyme undergoes; the second digit is the subclass, reflecting the type of bond breakage or formation taking place; the third digit represents the sub-subclass, which provides information on the chemical group involved in the enzymatic reaction; and the fourth level indicates the substrate specificity of the enzyme. The enzyme classification numbers of members in each FunFam were compared both at the 3-digit (EC3) and 4-digit (EC4) levels. The number of different EC codes among the relatives within a KinFam gives a measure of the functional purity of that kinase functional family.

For each KinFam, we calculated the information content of the multiple sequence alignment (MSA). This is captured as a diversity of position score (DOPs score) using Scorecons [39]. A DOPs score above 70 is a good indicator of a high diversity in the sequences. For FunFams with sufficient information content (DOPs > 70), Scorecons was also used to calculate the residue conservation at each position in the MSA. Previous analyses have shown that highly conserved residues in a FunFam are enriched in known functional residues (e.g., catalytic, ligand binding or protein interface residues) [26]. Thirty-two percent of the KinFams have a high DOPs score (>70). FunFams with low DOPs either contain very few sequences (<6) or are very species specific and lack diverse sequences.

As KinBase only provides sequences using their internal naming scheme, in order to extract Enzyme Commission codes (EC) we mapped KinBase entries to UniProt using BLASTP [40] to retrieve matches with 100% sequence identity.

### 2.5. Mapping of the CATH KinFams and KinBase Classifications

To compare the predicted CATH KinFams with the curated KinBase family classification, sequences from each KinBase family and subfamily were scanned against the KinFams-HMMs library using HMMER3 [24] with an e-value cutoff of 1 × 10^−18^ to give a mapping between the classifications. 

### 2.6. Mapping Drug Information from ChEMBL to Human KinFams

We previously developed a protocol [29] which associated domain families with drugs, by calculating the over-representation of drug targets within domain families. To identify druggable KinFams associated with human protein kinases, we adopted a similar approach: an FDA-approved kinase-inhibitor drug dataset was extracted from ChEMBL release 30 [41], https://www.ebi.ac.uk/chembl/, accessed on 2 November 2022). A drug was considered as a small molecule with therapeutic application, with direct binding to a single protein (ASSAY-TYPE = “B”), with a maximum phase of development = “4”, which indicates that the drug has been approved. Those with weak activity were filtered out by only considering a drug-target activity stronger than 1mM and a pChEMBL value of 6. The pChEMBL value is the measure of the half-maximal potency/affinity on a negative logarithmic scale. The anatomical therapeutic code (ATC-code) was used to select drugs that are protein kinase inhibitors. The ATC code classifies drugs into different groups at different levels (https://www.whocc.no/atc_ddd_index/, accessed on 24 January 2023). The code “L01E” corresponds to antineoplastic drugs which are protein kinase inhibitors.

### 2.7. Obtaining 3D Structures (PDB and AlphaFold2) for Human-KinFams

For all the sequences in human associated KinFams, we extracted the kinase domains from the PDB (https://www.rcsb.org/, accessed on 23 November 2022 [42]) or from the AlphaFold Protein Structure Database (https://alphafold.ebi.ac.uk/, accessed on 23 November 2022 [43]), as a 3D-model based on the sequence region of the functional unit in the UniProt sequence. We removed all AlphaFold2 models that did not fulfil the internal quality criteria established in Bordin et al. 2022 [44], which filters models based on below- pLDDT score > 70, more than 3 secondary structural elements, less than 65% of residues not in secondary structures, less than 30% of residues in long unordered regions, core packing and globularity.

We examined the extent of the structural diversity within kinase functional families within each human KinFam, by doing all-against-all structure comparisons of domain structures using the Sequential Structure Alignment Program (SSAP) [45].

## 3. Results and Discussion

### 3.1. Updating the CATH Kinase Superfamily

**Figure 3:**
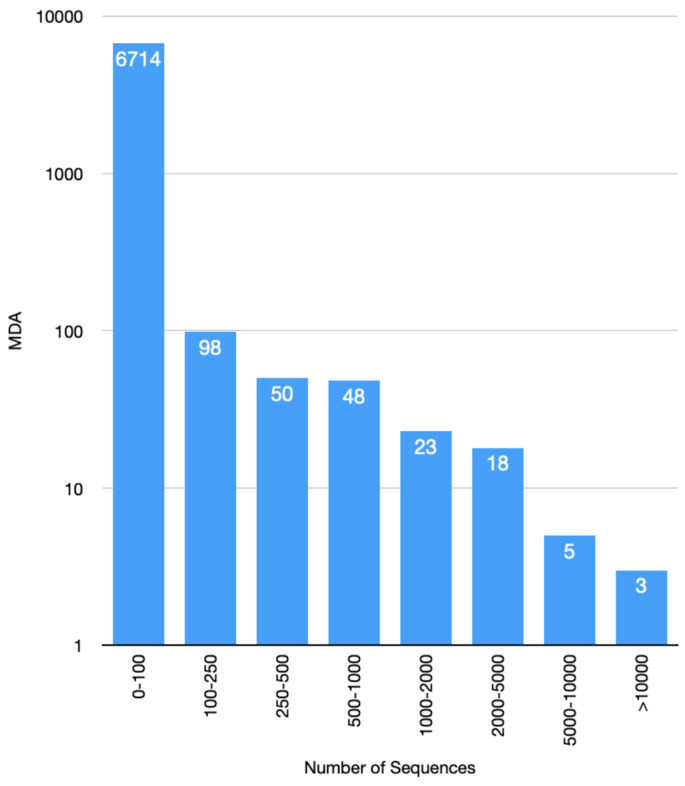
CATH kinase sequence distribution by multi–domain architecture (MDA).

Following the update of the CATH kinase domain superfamilies with sequences from UniProt (version release 2018_02) and concatenating N- and C- lobe sequences, 330,085 kinase functional unit sequences were obtained. As reported by Martin et al. [17], many kinases are multi-domain proteins and there is considerable diversity in their architectures, i.e., in the nature and order of domains in the protein sequence. Sequence distribution by MDA, is shown in Figure 3. There are 245 MDAs (out of 6958) comprising 100 sequences or more (see Figure 3). 

**Figure 4:**
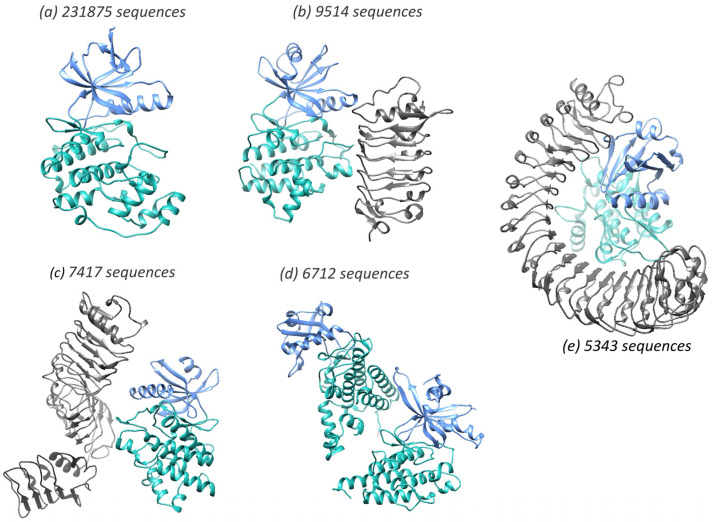
The most populated kinase multidomain architectures containing >5000 kinase sequences are shown. N-lobe is shown in blue, and the C-lobe is shown in cyan. The accessory domain is shown in grey. The domain architectures are illustrated using Alphafold2 structures from the following UniProt entries- (**a**) L7I0P6, (**b**) A0A445ETT0, (**c**) A0A178WEY8, (**d**) A0A444WN80 and (**e**) Q6XAT2.

The majority of MDAs are associated with small numbers of sequences whilst the largest 100 MDAs comprise 86.7% of the total sequences. The topmost populated domain architectures, in terms of number of sequences, are illustrated in Figure 4. It is worth noting that ~50% of the kinase sequences possess only the fused canonical N-C architecture (3.30.200.20-1.10.510.10) (Figure 4a). 

Within each of the 245 different MDA groups, kinase sequences were first clustered using CD-HIT at a 90% sequence similarity, resulting in 12,392 starting clusters across all MDAs. There were 39 clusters without experimental GO annotation that were not included in the FunFam generation but subsequently scanned against FunFams to identify the closest GO-annotated FunFams. The FunFam-MARC protocol was applied (see Methods) generating 2210 Kinase FunFams referred to as KinFams. Our KinFams contained a total of 330,085 sequences, a more than 40-fold increase over the number of sequences currently provided by KinBase.

**Figure 5:**
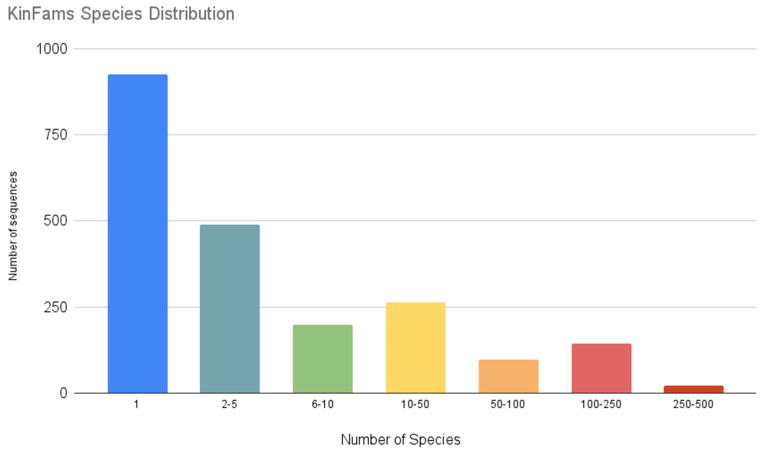
Species distribution associated with KinFams.

The majority of KinFams are organism-specific while a few KinFams represent sequences from more than 250 species (Figure 5).

### 3.2. Assessing the Functional Coherence of the KinFams

**Figure 6:**
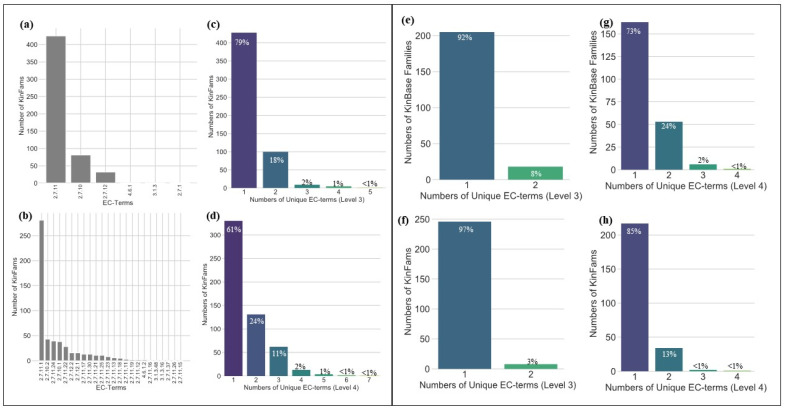
Distribution of EC-terms at levels 3 (**a**) and 4 (**b**) found in the KinFams for the complete set of 330,085 sequences classified in KinFams. Numbers of KinFams with one or more EC at level 3 (**c**) and EC at level 4 (**d**) in KinFams. It can be seen from Figure 6c,d that the majority of KinFams are associated with one EC3 and one EC4 term. For a subset of sequences in KinFams that map to KinBase, the right panel of the figure compares the numbers of unique EC terms at level 3 for KinBase (**e**) and KinFams (**f**) and level 4 for KinBase (**g**) and KinFams (**h**).

We analysed a subset of 543 KinFams comprising a total of 124,000 sequences (37.6% of the total kinase sequences), as these KinFams had one or more relatives with an experimentally characterised EC classification. We determined the number of EC terms in each KinFam, considering enzyme classification (EC) numbers at both the 3-digit and 4-digit EC-levels. Kinase sequences fall into 24 EC4 classes. Figure 6a,b shows the number of KinFams that fall into the different EC3 and EC4 terms assigned to kinase sequences. Figure 6c,d shows the number of EC terms assigned to KinFams. 

We compared the functional coherence of our KinFams classification with the KinBase classification using the same approach of considering the number of unique EC terms in each KinBase family and subfamily. We compared with KinBase because this resource was manually curated using experimental annotations for the sequences and is one of the most widely used and highly cited kinase classifications available. To make this comparison, we mapped KinBase sequences to KinFams to identify equivalent sequence sets (see Methods). It can be seen from Figure 6 that KinFams are more functionally coherent than KinBase subfamilies; the majority (85%) having only one EC4 term, compared to 73% for the KinBase classification. At the EC3 level, both KinFams and KinBase classifications have most families annotated with only one EC3 term 97% of relatives in KinFams and 92% of relatives in the KinBase classification have only one EC3 annotation. 

**Figure 7:**
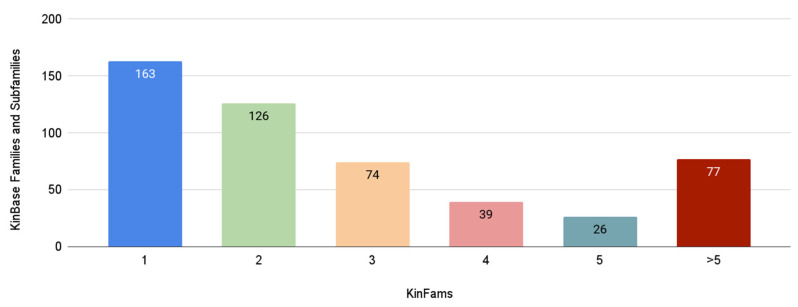
Mapping of KinBase to KinFams. The figure illustrates how many KinBase families and subfamilies are split into one or more KinFams by the FunFam-MARC protocol.

The improvement in EC functional coherence in KinFams was associated with the splitting of some KinBase subfamilies by the FunFam-MARC protocol. Figure 7 shows that 163 KinBase families and subfamilies have one-to-one mapping with KinFams, while 342 are split into two or more KinFams.

For certain KinBase groups and families, a further level of subclassification in subfamilies is not available. For a subset of these KinBase groups and families, KinFams is able to capture finer granularity in function by expanding the number of sub-families. 

**Figure 8:**
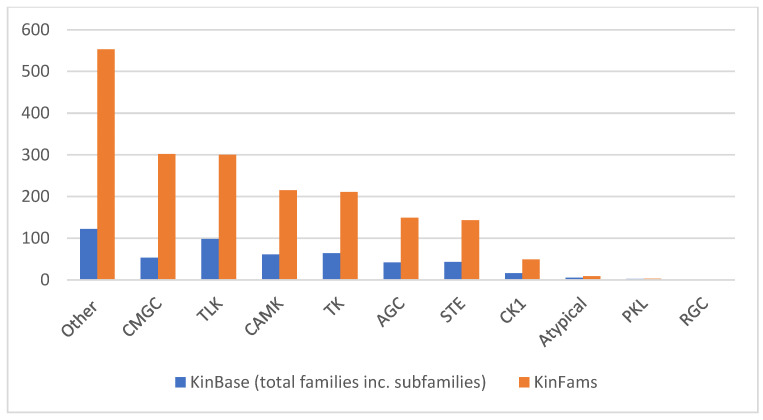
Group-wise expansion of subfamilies in KinFams, as compared to KinBase.

The group-wise expansion in the number of sub-families in KinBase due to KinFams is shown in Figure 8. The highest expansion (~5-fold) of family space in KinFams is observed for the KinBase ‘Other’ group. For the other kinase groups, the expansion varies from about 1.5-fold in case of two groups (Atypical and PKL) to about 3-fold expansion in the case of six groups (CMGC, TLK, CAMK, TK, AGC, STE and CK1). No expansion is seen in the case of the RGC group.

Whilst our EC analyses of the KinBase classifications suggested that a majority (73%) are likely to be functionally coherent, only a small proportion of KinBase sequences (11%) are experimentally annotated and therefore the subclassification of KinBase families by our KinFam protocol could reflect the detection of differences in Specificity-Determining Positions (SDPs). Below, we provide some examples illustrating the ability of our strategy to detect functional differences in relatives within KinBase families based on conservation of SDPs.

### 3.3. Example Illustrating KinFam Sub-Classification of the KinBase JAK Family 

The TK:Jak KinBase family includes the genes coding for the JAK1, JAK2, JAK3 and TYK2 proteins. These proteins comprise two distinct types of kinases, one of which is known to be catalytic and the other is reported as a pseudokinase (i.e., involved in non-catalytic, regulatory functions) [46]. For example, human JAK1 (UniProt ID: P23458) proteins are all annotated as EC 2.7.10.2. They comprise two kinases: the non-catalytic kinase (residues 583-855) and a catalytic kinase (residues 875-1153). Our protocol correctly subclassifies these into two distinct KinFams, namely KinFam-101 (catalytic) and KinFam-104 (non-catalytic, i.e., pseudokinase). 

**Figure 9:**
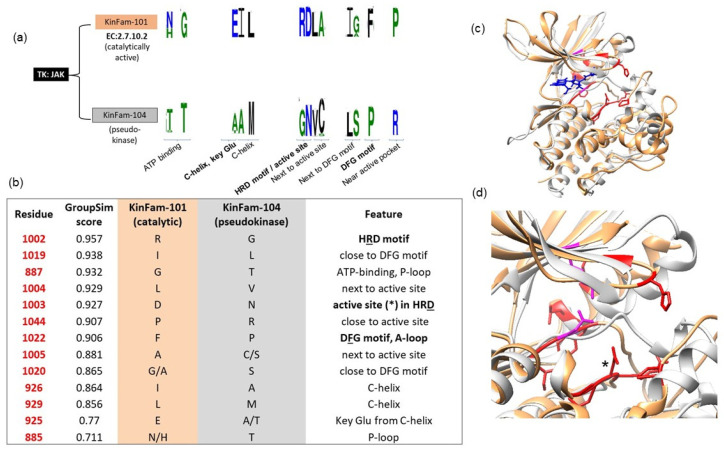
Specificity-determining positions (SDPs) predicted using CATH-KinFams: an example using the TK: JAK family. (**a**) TK: JAK family from KinBase is subdivided into two KinFams using CATH, each representing distinct kinases (catalytic and non-catalytic). (**b**) List of top-ranked SDPs (red) that are specific to each KinFam: 101 and 104: SDPs occur at/near the active site (within HRD motif in catalytic loop), at the DFG motif of the activation loop (residue numbering is shown according to the active kinase of JAK1, KinFam-101, PDB:6W8L). (**c**) Superposition of structures of the representatives of the catalytically active (orange, PDB: 6W8L, domain 875-1153) and pseudokinase (grey, AF_P23458, domain:583-855) of JAK1 (UniProt id: P23458). SDPs are shown in red. Ligand molecule from PDB:6W8L (namely R4S, which binds at the ATP-binding site), is shown in blue. (**d**) Close-up view of SDPs and their location within the catalytic and activation loops. Active site residue (D1003) in HRD motif is shown as asterisk. The other two active site residues are D1022 (from DFG motif) and K908 in the N-lobe (shown in magenta).

Our SDP analyses (see Figure 9) clearly indicate a variation between these two KinFams in several crucial sites of the kinase: the key HRD motif in the catalytic loop (RD is substituted with GN in the non-catalytic kinase) and within the DFG motif of the activation loop (F to P in the non-catalytic domain). Moreover, the C-helix E925, in the active kinase, which is in contact with the key active site K908 of the B3-strand (in the N-lobe), is equivalent to A/T638 in the pseudokinase. The salt bridge between the glutamic acid and lysine is crucial for the formation of the activated conformation of the kinase, as well as binding ATP, and the mutation might be partially responsible for the inactivity of the pseudokinase. 

The impacts of these mutations have been previously discussed in the literature [47], in a study which also highlights the lack of crucial autophosphorylation sites in the A-loop of the pseudokinase. Our SDP analysis, based on the KinFam classification, identifies further possible sites responsible for the inactivity of the pseudokinase, such as those involved in ATP binding (next to the DFG motif and the active site), as well as sites near the active site pocket: proline 1044, in contact with the active site, is mutated to an arginine in the pseudokinase, which may prohibit the ATP or phosphorylation substrate from entering into the active site pocket. The position of the SDPs is shown on Figure 9 below.

### 3.4. KinFam Subclassification of the KinBase HIPK Subfamily

HIPK (homeodomain-interacting protein kinase) is a subfamily belonging to KinBase family DYRK and the group CMGC. The HIPK subfamily comprises co-repressors that differentially interact homeodomain transcription factors [48].

The KinBase subfamily HIPK is divided by the FunFam-MARC protocol into two KinFams-10 and 319. KinFam-10 contains vertebrate HIPK1, HIPK2 and HIPK3 proteins (which share more than 90% sequence identity with each other). These are primarily present in the nucleus and expressed in all tissues [49]. By contrast, KinFam-319 consists solely of HIPK4 proteins, which occur in cytoplasm and are expressed mainly in testis and brain. The classification of HIPK4 into a distinct KinFam, is consistent with the fact that the HIPK4 protein is known to be a distant member of the KinBase HIPK family (sharing only 50% sequence identity with other HIPK1-3). In contrast to other HIPKs [1,2,3], HIPK4 occurs in the cytoplasm and lacks a nuclear localisation sequence and homeobox-interacting domain [49,50,51,52]. Additionally, in vitro studies confirmed that HIPK4 plays a unique role in regulating phosphorylation of manchette protein RIMBP3 during spermiogenesis [53]. A recent genome-wide microarray study suggested that HIPK4 does not primarily act through transcriptional control (unlike other HIPKs1-3), and that HIPK4 is essential for acrosome–acroplaxome function and male fertility [54]. The growing evidence from various experimental studies thus supports a distinct functional role of HIKP4 and endorses assignment to a distinct KinFam (KinFam-10), compared to the other HIPK1-3 proteins (KinFam-319).

**Figure 10:**
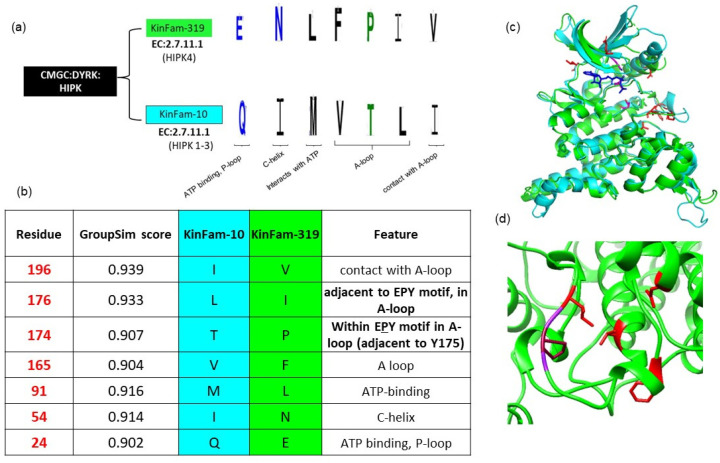
Specificity-determining positions (SDPs) predicted using CATH-KinFams: an example using the HIPK family. (**a**) HIPK family from KinBase is subdivided into two KinFams using CATH, each represents a distinct set of HIPK proteins (**b**) list of top-ranked SDPs (in red) that are specific to each KinFams-10 (HIPKs 1-3, cyan) and KinFam-319 (HIPK4, green). The majority of SDPs occur at and near the activation loop. SDPs (red) are numbered and mapped according to the AlphaFold2 af_Q8NE63_model. (**c**) Superposition of representative structures from KinFam-10 (PDB: 6P5S, green) and KInFam-319 (af_Q8NE63_model, HIPK4, cyan), respectively. Active site residues (K40, D136, D158) are shown in magenta; Ligand molecule (namely 3NG), which binds at the ATP binding site is shown in blue. (**d**) Close-up view of SDPs (in red) and their location within the ATP-binding site and activation loops.

Our SDP analysis shows that the majority of differentially conserved residues occur within the ‘activation loop’, that harbors the tyrosine residue required for autophosphorylation of HIPKs (Figure 10). This is particularly interesting because catalytic activity and subcellular localization of HIPKs is observed to be dependent on tyrosine autophosphorylation in the activation loop [49]. 

The activation loop of the DYRK family has a characteristic YxY element, whose second tyrosine is auto phosphorylated for kinase activation [55]. This motif is known to be altered to STY and EPY in HIPK1-3 and HIPK4, respectively. Interestingly, most of the SDPs are observed at and near this tyrosine-containing motif (See Figure 10). Additionally, E24Q substitution is observed in the P-loop lining the ATP-binding pocket, which also forms an interaction with an active site residue in the N-lobe. In summary, we identified additional SDPs within the activation loop, which are likely to be associated with distinct functional phenotypes in the HIPKs and which can suggest further investigation using experimental studies.

### 3.5. Merging of KinBase Groupings by KinFams

**Figure 11:**
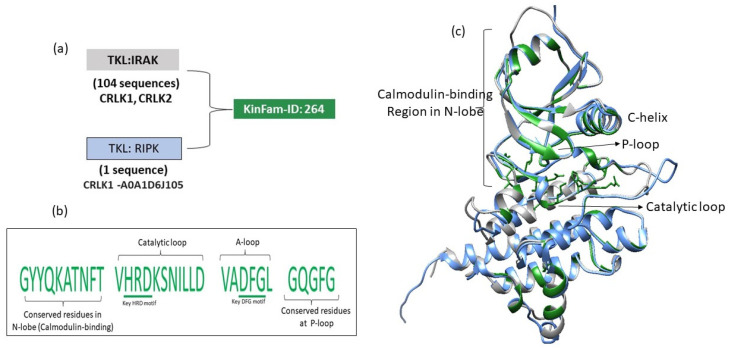
An example illustrating the merging of members of distinct families by the FunFam-MARC protocol. (**a**) KinFam-264 comprises 104 sequences from the KinBase TKL-IRAK family including calcium/calmodulin-regulated receptor-like kinases from plants (CRLK1 and CRLK2, e.g., UniProt ID: Q9FIU5). KinFam-264 merges a singleton sequence from TKL-RIPK, i.e., CRLK1 from *Zea Mays* [UniProt ID: A0A1D6J105]. (**b**) Closer inspection of conserved sites (shown in green) identified by Scorecons [39] indicates that many (91%) of the highly conserved residues (sites with Scorecons ≥ 90) in the larger group were shared by the singleton sequence. Conserved sites that are in the key functional regions are indicated in the figure-b. (**c**) The conserved sites are depicted using alphafold2 structures from TKL: RIPK (UniProt ID: A0A1D6J105, blue), and from the representative from TKL: IRAK (Q9FIU5, grey). The key regions are annotated. The majority of conserved sites are located in the N-lobe (known to harbor the calmodulin-binding site), the catalytic loop, the activation loop and the substrate binding site.

In some cases, the FunFam-MARC protocol merges distinct KinBase groupings into a single KinFam. The majority (82%) of the KinFams (73% of the sequences) map to a single KinBase family or subfamily. However, 18% of KinFams (comprising 4% of the total kinase sequences in KinFams) contain sequences from two or more KinBase subfamilies, whilst 11% of KinFams (2% of sequences) merge sequences from KinBase families and 4% of KinFams (0.3% of sequences) merge sequences from KinBase Groups (Figure 11, Table S3). This suggests that KinFams may sometimes miss subtle variations, for example between closely related species. It may also reflect the fact that the KinBase manual curation exploited other information besides sequence data, e.g., tissue specificity. 

We examined some of these cases and observed that most of the time, the protocol was merging a single sequence with a much larger set of KinBase sequences and that many of the highly conserved residues in the larger group were shared by the singleton sequence (see Figure 11). Our protocol exploits information on differentially conserved positions to segregate functionally distinct relatives. However, when one of the FunFams is very small (i.e., having few sequences) it can be difficult to determine the highly conserved positions unless the sequences are from very distant species. 

### 3.6. Increase in Kinase Family Space in KinFams Relative to KinBase

Our scans bring in protein kinases from all kingdoms and cover a total of 34,475 unique taxa (i.e., species). There is a 5-fold increase in the coverage of human kinase sequences relative to KinBase (2666 human domains in KinFams vs. 530 in KinBase). Out of 1,660,849 UniProt sequences assigned to KinFams, 47,359 (~3%), were annotated as putative or uncharacterised proteins in UniProt, so classification in KinFams is providing putative functions for these proteins based on the GO experimental annotations for the matched KinFam. 

Our KinFam classification identifies many more functional subfamilies than KinBase. Whilst some of these families may relate to a finer subclassification of KinBase families based on SDPs, some are likely to be novel families (see also below). Sequences from a more recent version of UniProt (release 2022_03) were scanned against the KinFam HMMs and sequences with an e-value below 1e-18 (threshold chosen to ensure functional similarity) were denoted as matches (see Materials and Methods), resulting in 1,790,576 matches from 1,660,849 UniProt entries, since some proteins contain more than one kinase domain.

A total of 505 (out of 579) KinBase families (i.e., 208 families and 297 subfamilies) map to 969 KinFams (out of 2210 KinFams). The remaining 74 KinBase families were not mapped to any KinFams as they were small or single sequence families, and the sequences are no longer maintained by UniProt. A further 1215 out of 2210 KinFams, are putative novel families comprising sequences not classified in KinBase. However, these KinFams appear to be functionally close to a KinBase family, i.e., they match the HMM for that family with an E-value of 10^−18^. This threshold has been suggested in previous studies to be associated with the functional similarity in catalytic mechanism and may be associated with some similarity in specificity. The remaining KinFams (26/2210) are outside the 1 × 10^−18^ (sequence similarity space) from a KinBase family and are therefore more likely to be completely novel families. Where experimental functional annotations are available for these potentially novel families, they are provided in Supplementary Table S1. 

### 3.7. Identifying Druggable KinFams

Since human kinases have been implicated in several diseases including cancer, we mapped clinically approved drugs to human KinFams. Sixty-one out of 246 human KinFams have relatives that are associated with drugs and diseases using data from ChEMBL version 30 [41] for drugs and from UniProt-Disease (https://www.uniprot.org/help/involvement_in_disease, accessed on 2 November 2022) for diseases.

**Figure 12:**
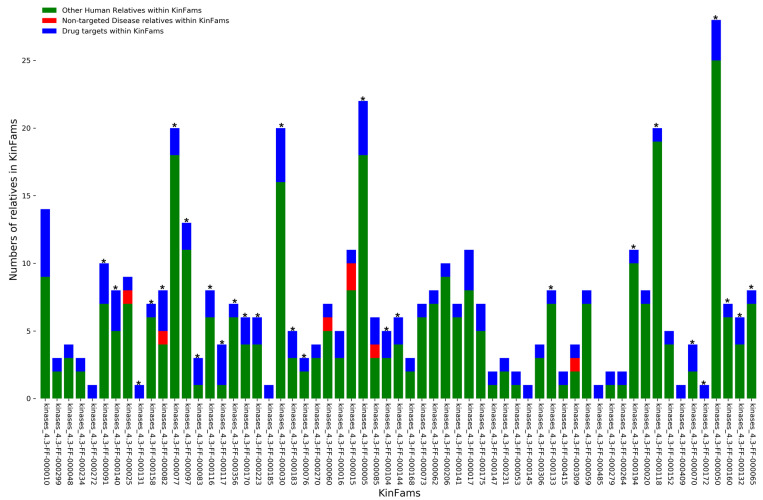
KinFams association with drug targets, i.e., 28 druggable KinFams based on overrepresentation of drug targets in the KinFam (shown as asterisk).

Kinases represent the second most targeted superfamily after the GPCRs, and they have the ability to provide novel usage of drugs to families associated with diseases which may help in repurposing available drugs. Therefore, we focused on identifying further druggable KinFams by means of a statistical analysis we used previously [29]. This connects drug with protein families, based on the statistical overrepresentation of the targets of the drug among the relatives of a protein family. We identified 28 druggable KinFams (Figure 12), that were associated with 47 drugs (BH False discovery q-value < 0.05) (See details in Supplementary Table S2).

Our analysis of the druggable KinFam shows a multi-drug association of drug compounds with the numbers of drugs associated with KinFams, ranging from 1–7. Most of the approved drugs associated with the KinFams are antineoplastic drugs (i.e., they prevent the growth of new tissues that may become cancerous). For example, the KinFam (“kinases_4.3-FF-000030”) is associated with the drugs ceritinib (CHEMBL2403108; used for the treatment of non-small cell lung cancer), ponatinib (CHEMBL1171837; developed for the treatment of chronic myeloid leukemia) and nintedanib (CHEMBL502835; used for some types of non-small-cell lung cancers). 

We have previously shown that relatives within the same druggable CATH FunFams are structurally conserved and have high conservation in the drug binding site [29]. Therefore, knowledge of the drug binding site in one or more of the relatives in a druggable KinFam may be useful for inheriting drug binding information to other relatives [56]. 

We already have experimental Protein Databank (PDB) structures for some relatives in some of the human KinFams. For the remaining sequences we extracted good quality models (see Methods Section 2.7) from the AlphaFold2 portal [43,57], where available. There are 246 human KinFams (comprising a total of 1379 sequences). Using the Alphafold2 and PDB domains associated with all sequences in these KinFams we performed and all-vs-all structural comparisons of relatives in each KinFam using SSAP [4]. We observed that for 80% of the human KinFams (75% of druggable KinFams) there is a very high average structural similarity between the relatives (RMSD < 3 Å, SSAP structure similarity > 90 (out of 100). Therefore, these KinFams may be particularly valuable for inheriting information on the drug binding pocket.

Druggable KinFams in which relatives share considerable similarity in structure, may also be valuable to consider when narrowing down therapeutic targets for a disease condition. This could be further substantiated by carrying out molecular dynamic simulations to establish potential binding energies for the various drugs associated with a druggable KinFam. Furthermore, relatives in the KinFams could be further explored to suggest possible side-effects of the drugs. However, this is beyond the scope of this current paper.

## 4. Conclusions and Future Directions

We developed the FunFam-MARC protocol which considers the multi-domain architecture of protein kinases and specificity determining residues to classify kinases into 2210 distinct kinase functional families (KinFams). KinFams are observed to have a higher functional purity in terms of EC annotations than families in the widely used canonical KinBase classification. This is due to the fact that we subclassified many (67%) of the KinBase families into two or more distinct KinFams. Although some apparently pure KinBase families may be split unnecessarily, given the lack of experimental annotation in most KinBase families, it is difficult to determine the extent to which we might over-split families. For example, in mammals, there may be differences in kinase interactions and thus functional specificities of kinases in different tissues, leading to changes in SDPs and giving rise to new KinFams. By contrast we rarely merge KinBase families.

A major advantage of our KinFam classification is the functional coherence of our functional families, which ensures that relatives can be easily aligned to give robust multiple sequence alignments that can be further analysed to detect highly conserved residues likely to be associated with the specificity of the kinase. 

Our classification approach is based purely on protein sequence information and does not take account of any experimental information on the oligomerization state, or known functional properties of the proteins, including substrate specificity, activity or subcellular localisation. Whilst these annotations are publicly available for some kinases, they are not comprehensive and therefore currently not sufficient for large-scale automated classifications, similar to our KinFams resource. However, previous analyses have shown that our CATH-FunFam protocol tends to implicitly capture residues differentially conserved between relatives associated with different multidomain compositions or oligomerization states, i.e., residues involved in domain-domain or protein-protein interfaces [26,27]. FunFams can also capture residues involved in promiscuous or moonlighting functions of the enzyme [58]. Furthermore, since KinFams are built from sequences in UniProt, it is possible to use the Uniprot ID to obtain a range of additional structural and functional annotations available from other resources (e.g., GO, PDB, REACTOME) to examine the similarity of these properties across a KinFam. 

We provide a catalogue of protein kinase families (KinFams) comprising sequences available in UniProt version 2018_02. We also provide information on the predicted multidomain composition of each kinase sequence with information on CATH superfamily assignment for each domain so that users can determine all of the partner domains in the kinase, beside the functional unit. We also provide hidden Markov models (HMMs) generated for each of the KinFams using HMMer3 [30,59]. The comprehensive mapping of UniProt sequences to KinFams revealed that our kinase family space covers all available taxa in UniProt (release 2022) including eukaryotes, prokaryotes and viruses. 

We demonstrated the application of our previously developed protocol [29] to find druggable families in the set of human protein kinases. Some of the structurally uncharacterised human (30%) KinFams have AlphaFold2 models of very good quality (i.e., pLDDT > 90). For some of these (80.8%), the high structural similarity between relatives is further evidence of a high functional similarity and suggests that drug binding characteristics can be inherited across relatives. The high-quality models will also be valuable for determining whether disease associated mutations lie on or close to functional sites and could be a modifying function or whether they are buried in the protein and the mutation could be destabilising the protein. In a small proportion of cases, we merge two or more KinBase families, but our FunFam-MARC protocol rarely merges KinBase groups. 

We have provided information on the KinFams via our CATH-FunVar (Functional Variation) resource [16]. This was previously established to display cancer associated CATH FunFams enriched in driver mutations. Whilst we have provided an initial set of predicted structures for human KinFams, we also aim to bring in alphfold2 domains of high quality (pLDDT ≥ 90) for all UniProt sequences in KinFams. The human KinFam classification will also be made available through CATH-FunVar. Thirty-two percent of KinFams have high information content (DOPs > 70) for which we provide information on conserved residues. This data can aid the characterisation of functional sites involved in the specificity and mechanism of the kinase. Where possible, we provided information on the EC purity of the KinFam as measured based on available experimental EC annotations for the relatives. We also provided information on the KinBase families that map to the KinFam to highlight KinFams where we merged KinBase families. This will allow users to derive multiple sequence alignments for these families and verify the degree of likely functional coherence across the family, by analysing highly conserved sites shared by the majority of the relatives.

Our KinFam classification was generated to test the ability of our FunFam-MARC protocol to identify functionally distinct families in a highly populated evolutionary superfamily. Only sequence data has been used to generate the classification. 

In future, we will explore the value of using the predicted structural data now available from the AlphaFold2 portal to improve our FunFam classification protocol. We hope that our KinFam data will facilitate the study of this diverse and medically relevant superfamily and help guide other kinase classification schemes and the experimental targeting of kinases that are predicted to have novel specificities. 

## Figures and Tables

**Table 1:** The summary of existing kinase classification schemes and associated resources. Hanks and Hunter (1995) developed the first kinase classification scheme, which was expanded in 1997 by the Bourne group (Protein Kinase Resource) and Manning group (KinBase). KinBase (version 2014, shown in bold). Other groups subsequently applied these classification schemes to map sequences from other additional species. For example, KinG has mapped sequences from over 200 organisms to families in Protein Kinase Resource (PKR). PrOKiNO has mapped kinase sequences from 1321 species to families from KinBase (2012). More recently, CATH-KinFams, described in this study, maps sequences from 13,981 species to 2210 functional families, using the novel FunFam-MARC protocol. ^a^ KinFams consist of alignments of kinase domain sequences from 13,981 species (UniProt release 2018_02). ^b^ Using HMMs built from these KinFams ^a^, we detected hits to an additional 20,494 organisms (total 34,475) from the latest UniProt release (2022_03).

Year	Name of Family Classification/ Database	Number of Groups/Families/ Subfamilies	Organisms/ Version of Uniprot or Swissprot Used	Website	Reference
1995	Hanks and Hunter	5 groups, 55 subfamilies	Model organisms	Not available	[7,8]
1997	PKR—Protein kinase resource	9 groups, 81 families, 238 subfamilies	SwissProt (2004)	http://pkr.sdsc.edu/html/index.shtml (not unavailable), accessed on 24 January 2023	[9]
2002	KinBase	KinBase 2014 version 14 Groups, 240 families, 339 Subfamilies	15 organisms	http://www.kinase.com/kinbase, accessed on 24 January 2023	[10]
2004, 2010	KinG database	PKR (as above)	>2000 organisms UniProt (2019)	http://king.mbu.iisc.ernet.in/, accessed on 24 January 2023	[14]
2007, 2009	Kinomer v.1	8 groups from KinBase (2008)	43 eukaryotic organisms	http://www.compbio.dundee.ac.uk/kinomer/index.html, accessed on 24 January 2023	[13]
2011, 2015	PrOKiNO	KinBase (2012 version) 14 groups, 273 Families and 359 Subfamilies	1321 organisms UniProt (2021)	http://vulcan.cs.uga.edu/prokino/about/prokino, accessed on 24 January 2023	[15]
2022	KinFams (CATH v4.3)	2210 KinFams	^a^ 13,981 organisms (from UniProt 2018) ^b^ 34,475 organisms (from UniProt 2022)	https://www.cathdb.info/, accessed on 24 January 2023	[16]

## Data Availability

The data generated in this study is made available through Zenodo (https://zenodo.org/record/7575924, accessed on 24 January 2023) and the CATH FTP (ftp://orengoftp.biochem.ucl.ac.uk/kinfams, accessed on 24 January 2023). Additional information on EC codes, GO terms and links to UniProt will be made available on CATH-FunVar (https://funvar.cathdb.info/, accessed on 24 January 2023).

## Supplementary Materials

The following supporting information can be downloaded at: https://www.mdpi.com/article/10.3390/biom13020277/s1, Table S1: Kinfams outside E-18, Table S2: 28 druggable KinFams in humans, Table S3: KinFams to KinBase mapping.
